# Influence of non-osteoporotic treatments in patients on active anti-osteoporotic therapy: evidence from the OSTEOMED registry

**DOI:** 10.1007/s00228-023-03544-x

**Published:** 2023-07-29

**Authors:** María Begoña Coco-Martín, Luis Leal-Vega, José Antonio Blázquez-Cabrera, Amalia Navarro, María Jesús Moro, Francisca Arranz-García, María José Amérigo, Manuel Sosa-Henríquez, María Ángeles Vázquez, María José Montoya, Manuel Díaz-Curiel, José Manuel Olmos, Marta Ruiz-Mambrilla, José Filgueira-Rubio, José Luis Pérez-Castrillón, on behalf of the OSTEOMED Group, José Filgueira-Rubio, Nerea Hernández-de Sosa, María Luz Calero-Bernal, Dolors Armengol-Sucarrats, Begoña de Escalante-Yanguas, Cristina Miranda-Díaz, María José Miranda-García, Mercedes Giner-García, Julia Jareño-Chaumel, Rafael Cotos-Canca, José Luis Hernández, Francisco Javier Rodero-Hernández, Pilar Sánchez-Molini, José María Aguado-Caballero, Juan Carlos Cobeta-García, Raimundo Tirado-Miranda

**Affiliations:** 1 Group of Applied Clinical Neurosciences and Advanced Data Analysis, Department of Medicine, Dermatology and Toxicology, University of Valladolid, Valladolid, Spain; 2 Department of Internal Medicine, Hospital General Universitario de Albacete, Albacete, Spain; 3 Department of Internal Medicine, Hospital Universitario Infanta Leonor, Madrid, Spain; 4 Department of Internal Medicine, Hospital Clínico San Carlos, Madrid, Spain; 5 Department of Internal Medicine, Hospital Universitario Insular de Gran Canaria, Las Palmas de Gran Canaria, Las Palmas, Spain; 6 Department of Medicine, University of Seville, Seville, Spain; 7 Department of Internal Medicine, Fundación Jiménez Díaz, Madrid, Spain; 8 Department of Internal Medicine, Hospital Universitario Marqués de Valdecilla, Cantabria, Spain; 9 Unit of Speech and Language Therapy, Department of Surgery, Ophthalmology, Otorhinolaryngology and Physical Therapy, University of Valladolid, Valladolid, Spain; 10 Department of Internal Medicine, Hospital Viamed Santa Elena, Madrid, Spain; 11 Department of Internal Medicine, Hospital Universitario Río Hortega, Valladolid, Spain

**Keywords:** Osteoporosis, Fractures, Prescription drugs, Cohort analysis, Logistic models

## Abstract

**Purpose:**

To evaluate the effect of different non-osteoporotic drugs on the increase or decrease in the risk of incident fragility fractures (vertebral, humerus or hip) in a cohort of patients diagnosed with osteoporosis on active anti-osteoporotic therapy.

**Methods:**

For this retrospective longitudinal study, baseline and follow-up data on prescribed non-osteoporotic treatments and the occurrence of vertebral, humerus or hip fractures in 993 patients from the OSTEOMED registry were analyzed using logistic regression models. The drugs evaluated with a possible beneficial effect were thiazides and statins, while the drugs evaluated with a possible harmful effect were antiandrogens, aromatase inhibitors, proton pump inhibitors, selective serotonin reuptake inhibitors, benzodiazepines, GnRH agonists, thyroid hormones, and oral and inhaled corticosteroids.

**Results:**

Logistic regression analyses indicated that no treatment significantly improved fracture risk, with the only treatments that significantly worsened fracture risk being letrozole (OR = 0.18, *p*-value = 0.03) and oral corticosteroids at doses ≤ 5 mg/day (OR = 0.16, *p*-value = 0.03) and > 5 mg/day (OR = 0.27, *p*-value = 0.04).

**Conclusion:**

The potential beneficial or detrimental effects of the different drugs evaluated on fracture risk are masked by treatment with anabolic or antiresorptive drugs that have a more potent action on bone metabolism, with two exceptions: letrozole and oral corticosteroids. These findings may have important clinical implications, as patients receiving these treatments are not fully protected by bisphosphonates, which may imply the need for more potent anti-osteoporotic drugs such as denosumab or teriparatide.

## Introduction

Osteoporosis is the most common metabolic bone disease, defined by a decrease in bone strength that predisposes to the development of fragility fractures [1, 2]. These constitute the most serious complication of the disease, with a high morbidity and mortality rate [3].

Several modifiable and non-modifiable factors are involved in its occurrence, such as age, sex, genetic factors, bone mineral density (BMD), previous fractures, comorbidities and prescription drug intake [4]. Among the non-modifiable factors, age is one of the most important, as older age is associated with a greater number of comorbidities and a higher intake of drugs not specifically used to treat osteoporosis [5].

Several clinical studies carried out in cohorts of patients have shown that some of these drugs can increase or decrease the risk of fractures. This has led to the individualization of treatment, so that those patients with a higher risk of fractures should receive treatments with a neutral or beneficial effect on bone metabolism, and those that are harmful should be avoided. Most of these studies have been conducted in general population cohorts or in cohorts of patients characterized by underlying disease (hypertension, dyslipidaemia, diabetes, inflammatory diseases, etc.), with few studies evaluating this effect in patients diagnosed with osteoporosis on active treatment [6–10].

The aim of this study is therefore to evaluate the effect of different drugs on the increase or decrease in the risk of incident fragility fractures (vertebral, humerus or hip) in a cohort of patients with osteoporosis on active anti-osteoporotic treatment.

## Methods

### Study design

This retrospective observational study examined whether taking different drugs increased or decreased the risk of incident fragility fractures after a follow-up period of ≥ 1 year in a cohort of osteoporotic patients on active anti-osteoporotic treatment.

To do this, we used data from the OSTEOMED registry, made up of patients who attended internal medicine consultations in 23 Spanish hospitals for the diagnosis or assessment of osteoporosis or the presence of fractures between 2012 and 2017 [11].

### Study population

The population of this study consisted of 993 patients from the OSTEOMED registry with matching baseline and follow-up data [912 women (91.84%), 81 men (8.16%), mean age of 65.39 ± 11.15 years]. Patients included in this registry were mainly referred to internal medicine consultations from primary care, other hospital departments and other internal medicine department consultations.

Patients diagnosed with osteoporosis according to the densitometric criteria established by the World Health Organization (WHO) (T-score < -2.5 at any location) or with typical fragility fractures (vertebral, humerus or hip) regardless of their BMD were included in the study. On the other hand, patients with malignancies, a life expectancy of < 1 year or aged > 90 years were excluded from the study, as their follow-up in the proposed manner was considered unfeasible.

This study has been approved by the Clinical Research Ethics Committee of the Albacete University Hospital Complex (Act 02/11) and has been conducted in accordance with the Declaration of Helsinki and its later amendments or comparable ethical standards.

Follow-up of the patients included was performed according to standard clinical practice, meaning that no additional diagnostic tests or therapeutic interventions were performed. However, all patients received an information sheet on the aims of the study and signed a written informed consent prior to clinical data collection.

### Study variables

The variables collected were from a medical record specifically focused on osteoporosis and fractures. Fractures and prescribed anti-osteoporotic and non-osteoporotic treatments were obtained from patients' medical records and then entered into a dedicated electronic database by trained research staff from the centres participating in the study.

Variables were collected at two visits, an initial visit when patients were first referred to the internal medicine consultation for the diagnosis or assessment of osteoporosis or the presence of fractures, and a follow-up visit after a minimum follow-up period of 1 year.

First, two numerical variables were created for the total number of vertebral, humerus and hip fractures recorded at baseline and follow-up, respectively. Importantly, all fractures were radiographically confirmed by a specialized physician.

A categorical variable called fracture variation was then created, which took the value of "0" when the total number of fractures between baseline and follow-up remained the same (improvement) and the value of "1" when the total number of fractures between baseline and follow-up increased (worsening).

Other categorical variables created were sex (male or female), age range (< 65, 65 to 75 or > 75 years), anti-osteoporotic treatment prescribed to patients, which took the value of “0” or “1” depending on whether patients were taking vitamin D, alendronate, risedronate, calcium, strontium, teriparatide or denosumab; and non-osteoporotic treatment prescribed to patients, which took the value of “0” or “1” depending on whether patients were taking antiandrogens (AA), aromatase inhibitors (AI), gonadotropin-releasing hormone (GnRH) agonists, selective serotonin reuptake inhibitors (SSRI), benzodiazepines, proton pump inhibitors (PPI), thyroid hormones, oral corticosteroids, inhaled corticosteroids, thiazide diuretics or statins.

### Statistical analysis

Different logistic regression models have been used for the purpose of this study. Logistic regression is a predictive modelling technique that provides a predictive model to explain a dichotomous dependent variable from independent variables. The function of the model is to predict the probability of belonging to a category or group (in our case the probability of decreasing the risk of fracture versus not decreasing the risk of fracture) based on the non-osteoporotic treatments evaluated in our cohort.

Another key measure calculated was the odds ratio (OR) associated with each treatment, which reflects how many times the probability of improvement in fracture risk is greater than the probability of worsening when received. The OR takes values between “0” and infinity. Values > 1 mean that the probability of improvement increases and values < 1 mean that the probability of improvement decreases. The more the OR exceeds “1”, the greater the likelihood of improvement with treatment.

The results of this study were obtained by analyzing the data with the statistical software package R 4.1.2.

## Results

### Population

The sex, age range and prescribed anti-osteoporotic treatment of the 993 patients included for statistical analysis are shown in Table 1.

**Table 1:** Sex, age range and anti-osteoporotic treatment prescribed to patients

Sex	
Females	912
Males	81
Age range	
< 65	492
65–75	292
> 75	209
Anti-osteoporotic treatment	
Vitamin D	808
Calcium	724
Strontium	48
Alendronate	103
Risedronate	139
Teriparatide	120
Denosumab	179

### Fractures

The total number of vertebral, humerus and hip fractures that patients had at baseline and follow-up is shown in Table 2.

**Table 2:** Fractures presented by patients at baseline and follow-up

Fractures	Baseline	Follow-up
Vertebral	41	42
Humerus	13	5
Hip	165	8
Total	219	55

### Non-osteoporotic treatment

The number of patients taking the different non-osteoporotic treatments evaluated in the study is shown in Table 3.

**Table 3:** Evaluated non-osteoporotic treatments that patients received

AA	1
AI	40
Anastrozole	19
Letrozole	12
Exemestane	9
PPI	113
SSRI	58
Benzodiazepines	97
GnRH agonists	2
Thyroid hormones	98
Oral corticosteroids	41
≤ 5 mg/day	10
> 5 mg/day	31
Inhaled corticosteroids	17
≤ 1000 µg/day	16
> 1000 µg/day	1
Thiazides	42
Statins	153

*AA* antiandrogens, *AI* aromatase inhibitors, *PPI* proton pump inhibitors, *SSRI* selective serotonin reuptake inhibitors, *GnRH* gonadotropin-releasing hormone

Using analysis of variance (ANOVA), we studied whether the mean number of fractures at baseline differed significantly from the mean number of fractures at follow-up, finding that it did not (*p*-value = 0.258, 95% CI). The paired sample t test used to test whether the mean number of fractures at follow-up increased from baseline showed a difference in means (*p*-value < 0.001), confirming that the mean number of fractures at follow-up was significantly lower than the mean number of fractures at baseline. The difference in means was also confirmed by the Wilcoxon signed-rank test (p-value < 0.001) and thus confirms that the risk of fractures decreased significantly between baseline and follow-up.

Logistic regression models used to explain the probability of improving fracture risk as a function of non-osteoporotic treatments prescribed to patients showed that no treatment significantly improved the risk of fracture, the only treatments that significantly worsened fracture risk being letrozole (OR = 0.18, *p*-value = 0.03) and oral corticosteroids at doses ≤ 5 mg/day (OR = 0.16, *p*-value = 0.03) and > 5 mg/day (OR = 0.27, *p*-value = 0.04).

The results of the logistic regression models used to find out which treatments improve or decrease the risk of fracture occurrence according to the *non-osteoporotic treatments* prescribed to the patients are shown in Table 4.

**Table 4:** Evolution of fractures according to non-osteoporotic treatments that patients received

Patients (n)	993	
Fracture risk improvement (n)	952	
Fracture risk improvement (%)	95.8%	
Correctly classified cases (%)	95.7%	
	OR	*p*-value
AA	N/A	0.99
AI		
Anastrozole	N/A	0.99
Letrozole	0.18	0.03*
Exemestane	N/A	0.99
PPI	1.15	0.79
SSRI	0.66	0.49
Benzodiazepines	0.53	0.19
GnRH agonists	0.80	1.00
Thyroid hormones	1.38	0.59
Oral corticosteroids		
≤ 5 mg/day	0.16	0.03*
> 5 mg/day	0.27	0.04*
Inhaled corticosteroids		
≤ 1000 µg/day	N/A	0.99
> 1000 µg/day	3.94	0.99
Thiazides	N/A	0.98
Statins	0.88	0.77

N/A: It was not possible to calculate the OR

*AA* antiandrogens, *AI* aromatase inhibitors, *PPI* proton pump inhibitors, *SSRI* selective serotonin reuptake inhibitors, *GnRH* gonadotropin-releasing hormone

*p-value < 0.05

## Discussion

Our results show that in this cohort of patients diagnosed with osteoporosis on active anti-osteoporotic treatment, drugs that may have a beneficial effect on fracture risk reduction (thiazide diuretics and statins) have a neutral effect. However, some drugs such as AI and oral corticosteroids may have a detrimental effect despite anti-osteoporotic therapy.

Thiazides exert a small, positive effect on BMD, which leads to a decrease in the risk of fractures. This effect disappears 4 months after withdrawal [12]. The mechanism of this effect is multifactorial. On the one hand, they have a positive effect on bone remodeling by inhibiting osteoclasts and increasing osteoblast bone-forming activity, and on the other hand, they facilitate calcium absorption by bone and increase its plasma concentration by increasing renal tubular reabsorption of calcium. This is associated with an inhibition of parathyroid hormone (PTH). The latter suppresses the production of RANKL, the main factor in the maturation and activation of osteoclasts, cells that increase bone resorption, leading to a decrease in bone quantity and quality [13]. However, in our population this reduction in bone remodeling is not perceived by the body as patients are receiving more potent antiresorptive agents. A similar situation occurs with statins, which act at the level of the mevalonate pathway, inhibiting it and thus decreasing cholesterol synthesis. This pathway is used by osteoclasts to produce the prenylation of small proteins necessary for the inhibition of osteoclast apoptosis. Specifically, there is a decrease in the synthesis of isoprenoids, farnesyl diphosphate and geranylgeranyl diphosphate, which are involved in the prenylation of GTPases that regulate various osteoclastic processes [14]. Therefore, although statins have a potential beneficial effect, as previously seen in non-osteoporotic patients, the use of potent anti-resorptive drugs appears to inhibit this effect [9].

In this study, AA, AI, PPI, GnRH agonists, SSRI, benzodiazepines, thyroid hormones and oral and inhaled corticosteroids have been evaluated as non-osteoporotic treatments that may increase the risk of fractures.

AA increase the risk of fracture by suppressing androgen production and have been used to improve bone health in cancer patients. GnRH agonists inhibit their production by the pituitary gland, decreasing ovarian estrogen production. This decline increases osteoclast activity, reducing bone strength and facilitating the development of fractures [15].

In turn, several studies have shown the detrimental effect of PPI on bone. Vestergaard et al. [16] in a case-control study with a very large number of patients observed an increased risk of fractures (OR = 1.18, 95% CI: 1.12–1.43), especially hip fracture (OR = 1.60, 95% CI: 1.25–2.04). This effect was attributed to decreased intestinal calcium absorption with secondary hyperparathyroidism and negative calcium balance.

The association between antidepressants and fracture has also been the subject of various studies of different natures (case-control, cohorts, etc.), observing that the use of tricyclic antidepressants and SSRI increase the risk of fractures. Depressed patients have a lower bone mass, which may increase this risk. In addition, 5-HT receptors are present in bone cells (osteoblasts, osteoclasts, osteocytes) regulating the bone neuroendocrine system, so their inhibition will increase the risk of fractures [17].

The use of benzodiazepines has also been associated with an increased risk of fractures, especially hip fractures [18]. For example, a meta-analysis aimed at evaluating the effect of zolpidem involving 1,000,000 individuals found an increased risk of fractures (OR = 1.92, 95% CI: 1.65–2.24), with an increased risk of hip fracture (OR = 2.8, 95% CI: 2.19–3.58) [19]. The mechanism is related to an increased risk of falls secondary due to adverse effects of the drug, such as drowsiness, unsteadiness and lack of coordination.

Thyroid hormone replacement therapy may also lead to an increased risk of fractures if normalization of function has not been achieved. Hypothyroidism causes an inhibition of bone remodeling, hindering the renewal of biomechanically defective bone and therefore decreasing bone strength [20], while hyperthyroidism in turn increases bone remodeling, decreases bone mass and increases the risk of fractures [21].

All the drugs discussed have a deleterious effect on bone health and increase the risk of fractures. However, in our study none of them have shown this detrimental effect as their effects on bone mass and remodeling are not intense and the use of potent antiresorptive drugs could counteract this effect, with two exceptions: letrozole and oral corticosteroids at doses ≤ and > 5 mg/day.

AI such as letrozole, the most commonly used, prevent the formation of estrogens from fat tissue. In postmenopausal women, these estrogens are derived from adrenal androgens through the action of the enzyme aromatase, which is highly expressed in breast and fat tissue ^15^. Its decline increases bone remodeling, which leads to a decrease in bone strength and predisposes to an increased risk of fractures. The lack of efficacy of bisphosphonates may be related to the fact that they begin to have an anti-fracture effect 6–12 months after the start of treatment. The follow-up period used in our study was ≥ 1 year so it is possible that their benefit cannot be observed. Previous studies have demonstrated the benefit of bisphosphonates in patients treated with AI [22]. However, in a systematic review which compared the efficacy of bisphosphonates and denosumab in patients treated with IA, it was found that both drugs increased BMD, but only denosumab reduced the occurrence of fractures [23]. These results are comparable to those observed in our study.

A similar effect could explain the lack of response in patients taking oral corticosteroids, given that these drugs exert their detrimental effect on bone through different endocrine and autocrine mechanisms, inhibiting bone formation and facilitating the risk of fractures. Corticosteroids reduce the number of osteoblasts and inhibit their functions and stimulate the production of osteoclasts and increase their activity. Stimulation of PPAR-γ facilitates adipocyte formation and inhibits Wnt/β-catenin pathway, inducing osteocyte apoptosis. They also increase hypogonadism and renal calcium loss, decrease physical activity, GH and IGF-1 and induce muscle atrophy [24]. These alterations increase the risk of fractures especially in the first 6 months of treatment, a period in which bisphosphonates are less effective (specifically alendronate, the most commonly used in our cohort) [6, 25, 26]. It should be noted that the detrimental effect of oral corticosteroids has been observed at both doses evaluated (≤ and > 5 mg/day). This fact may be in contradiction with clinical practice guidelines that recommend active treatment to prevent osteoporosis in patients receiving ≥ 5 mg/day [3]. However, this refers to the general population, not necessarily to patients with osteoporosis. Hence, the present study shows a detrimental effect of oral corticosteroids, regardless of dose, in patients diagnosed with osteoporosis on active anti-osteoporotic treatment, mainly bisphosphonates.

## Conclusion

This study evaluated the beneficial or detrimental effect of various drugs in a population of patients diagnosed with osteoporosis on active anti-osteoporotic treatment. The main outcome measure was the occurrence of incident fragility fractures (vertebral, humerus or hip) during a follow-up period of ≥ 1 year. In the "Discussion" section, we have analyzed the mechanisms of action of the different drugs evaluated on bone metabolism, finding that these effects are masked by treatment with anabolic or antiresorptive drugs that have a powerful action on bone remodeling. We have observed only two exceptions, letrozole, the most frequently used AI in the treatment of breast cancer, and oral corticosteroids in doses both ≤ and > 5 mg/day. These findings may have important clinical implications, as patients receiving these treatments are not fully protected with bisphosphonates, which could imply the need to prescribe more potent anti-osteoporotic drugs, such as denosumab or teriparatide, whose superiority in increasing bone mass and reducing fracture risk has been demonstrated in previous studies [27, 28].

The strengths of the present study are determined by the sample size, the method of data collection (protocolized electronic medical record) and the statistical analysis used, while the weaknesses are determined by the short follow-up period and the non-representation of certain drugs that may have a detrimental effect on bone remodeling.

In summary, the effect of the different drugs evaluated on the risk of fractures in patients diagnosed with osteoporosis on active anti-osteoporotic therapy is scarce. No beneficial effects were observed and only the use of AI such as letrozole and oral corticosteroids at doses ≤ and > 5 mg/day appear to have a detrimental effect on fracture risk.

## Data Availability

The datasets generated during and/or analysed during the current study are available from the corresponding author on reasonable request.
